# From the Editor

**DOI:** 10.34763/devperiodmed.20172103.175176

**Published:** 2017-10-28

**Authors:** Barbara Woynarowska

The increase of obesity in children and young people across the globe is becoming a challenge for the medicine of today. The task is very serious and difficult when taking into consideration the consequences of obesity: health problems (cardiovascular diseases, diabetes type 2 etc.), as well as psychosocial and economic implications. The origin and mechanisms underlying the rapid increase of obesity in children is complex and not very well known, therefore successful prevention and treatment are difficult. In this situation reguired is a multidisciplinary team ensuring good cooperation of different specialists in order to develop a programme of prevention involving a diagnosis and treatment procedure. As we have already stressed, it is a very difficult task and up to now the results achieved are not satisfactory enough.

In 2014 the European Commission launched the EU Action Plan on Childhood Obesity 2014-2020 in Europe, which is based on eight key areas for action: 1) Support a healthy start in life; 2) Promote healthier environments, especially in schools and pre-schools; 3) Make the healthy option the easier one; 4) Restrict marketing and advertising to children; 5) Inform and empower families; 6) Encourage physical activity; 7) Monitor and evaluate; 8) Increase research. The WHO European Food and Nutrition Action Plan 2015-2020 moreover included the task concerning the adoption of comprehensive intervention and community-based initiatives to improve nutrition and prevent overweight and obesity among pre-school and school-aged children.

Overweight and obesity in children and adolescents is the subject of ongoing research projects. Many strategies for coordination or integration and dissemination of research findings and turning them into innovative actions are undertaken at international level. Some examples of these activities are the European Childhood Obesity Group (ECOG) which was established in 1990, or the Child Obesity Task Force (COTF) formed within the framework of the European Association for the Study of Obesity (EASO) in 2000. A member of ECOG, Professor Marie-Laure Frelut was kind enough to write the editorial for this issue of Developmental Period Medicine presenting the progress and challenges in tackling child and adolescent obesity*In 2011 year whole number 3 of Developmental Period Medicine was dedicated to research publications concerning investigations on Maternal and Early Life Obesity.. In 2016 the WHO Regional Office for Europe established the Childhood Obesity Surveillance Initiative (COSI) in more than half the countries in the European Region. The aim of the Initiative is to measure trends in overweight and obesity in children aged 6.0-9.9 years to get a clear understanding of the epidemic and to allow inter-country comparisons. Data concerning the prevalence of overweight and obesity among 11-15-year-olds are collected every four years within the framework of Health Behaviour in School-aged Children (HBSC), a WHO collaborative cross-national study which has been in place since 2002. In the last survey (2013/14) 44 countries or regions participated in this study.

Also in Poland many initiatives on prevention, management and research concerning childhood obesity were undertaken. The Institute of Mother and Child participated in the HBSC study (some data are presented in two articles in this issue) and the COSI project. The Children’s Memorial Health Institute carried out a nationwide epidemiological study on the prevalence of overweight and obesity among children and adolescents aged 2-19 years (OLA and OLAF study). The Institute of Food and Nutrition carried out research and coordinated broad actions on obesity prevention among pregnant women, children and adolescents within the framework of the Project Supported by a Grant from Switzerland through the Swiss Contribution to the Enlarged European Union. In The National Health Programme 2016-2020 the first of its six operational aims is: “Improvement of nutrition, nutritional status and physical activity of society”. Many tasks included in this aim created the basis for childhood obesity prevention and therapy activities undertaken by different sectors.

In appreciation of the need for a multi-faceted approach to the prevention, management and therapy of childhood obesity, this edition of “Developmental Period Medicine” features a series of articles prepared by specialists from different disciplines. The articles can be divided into the following broadly conceived groups (some of them overlapping):

## Epidemiology

In the article written by A. Grajda et al., based on the results of the nationwide OLAF study, differences in the prevalence of overweight and obesity among children aged 6-19 years in different regions are analyzed. It was showed that in the region of Eastern Poland with GDP per capita below 80% of the national average, the prevalence of these disorders was lower (14.7%) in comparison with the rest of the country (16.4%).

## Determinants

### • Genetic

The genetics of obesity – its pathogenetic, clinical and diagnostic aspects are presented by A. Barczyk, E. Obersztyn et al. Based on the results of multi-center studies three types of genetically conditioned obesity are broadly described: isolated, monogenic obesity; syndromic monogenic obesity associated with dysmorphic features and/ or congenital defects caused by mutation in specific gene(s); chromosomal aberrations, including submicroscopic changes. The neuro-endocrinological regulation of hunger and thirst, the clinical consequences of mutation in genes associated with the melanocortin pathway and the features of the most common obesity syndrome, as well as the diagnostic algorithm for cases of suspected syndromic obesity is presented.

### • Nutrition

Appropriate dietary patterns in children determines their optimal development, therefore playing a very important role. The investigation conducted by H. Weker, M. Barańska et al has shown that the diet of overweight toddlers differs from the safe nutrition model in a very evident way. The solution is to implement nutritional education (H. Weker, M. Barańska et al).

### • Microbiota

Obesity and microbiota. The role and function of the gut microbiota in contributing to the pathogenesis of obesity and the metabolic syndrome is presented by A. Karney.

### • Psychology

Psychological determinants. The role of the mother-child relationship, specific characteristics of the relationships; children’s body experiences and certain body image distortions are described as psychological mechanisms involved in the onset and maintenance of childhood obesity (J. Radoszewska). A. Dzielska et al. present the Polish version of “The Physical Appearance Comparison Scale” (PACS), which may be used as a reliable and valid tool in the diagnosis and management of adolescents with excess body mass.

### • Physical activity

The lack of physical activity plays an evident role in the increase of obesity. Based on the literature review and own experiences W. Osiński and A. Kananista describe very useful practical recommendations regarding the planning, implementing, and monitoring of intervention programmes involving controlled physical activity aimed at the reduction of adipose tissue. The analysis of the data from the HBSC study presented by D. Kleszczewska et al. showed that the percentage of overweight 15-year-olds in Poland was lower than the international average, but the percentage of adolescents, especially girls, who consider themselves too fat, was much higher. The level of discrepancies between objective and subjective assessment of body mass depends on geographical and cultural differences, and the level of physical activity modifies these discrepancies. The perceived barriers to physical activity in adolescents and their association with motivation are presented by M. Jodkowska et al. It was found that three barriers (lack of energy, skills and willpower) and the perception of several barriers occurring simultaneously were reported more frequently by overweight adolescents than their peers with normal body mass. Motivation was a key element of perceiving these barriers.

## Consequences of obesity

Two papers present the metabolic consequences of obesity related mainly to increasing the risk of atherosclerosis and cardiovascular diseases and diabetes type 2. In the group of obese children dyslipidemia, high insulin level and carotid intima-media thickness (IMT) were higher than in the control group (A. Karney et al). The concentrations of low-density lipoprotein (LDL) and vitamin A in obese children were higher than in children with normal body mass (J. Gajewska et al).

## Therapeutics strategies

A combined mode of therapy of obese children and adolescents consisting of behavioral, dietary counseling (Dietary patterns in toddlers with excess weight 2016 Pitnats study – H. Weker, M. Barańska et al) with appropriate physical activity and using both modern media and devices are described by A. Zachurzok et al. It was stressed that successful therapy required good cooperation of the therapeutic team with the children and their parents.

The editorial board believes that the papers presented in the present issue will be interesting and useful for different professionals working with young obese people and will stimulate research in this field. An additional value are the lists of references included in the articles presented.

